# Supplementary material for: South African consumers’ perceptions of front-of-package warning labels on unhealthy foods and drinks
Source: PLoS One. 2021 Sep 27;16(9):e0257626. doi: 10.1371/journal.pone.0257626 (PMC8475997; doi:10.1371/journal.pone.0257626)
Supplement: S1 Fig — (PDF) [file pone.0257626.s001.pdf]

Saturated fats icons (Yoghurt)

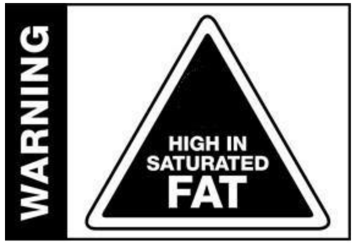

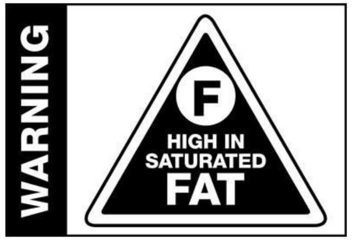

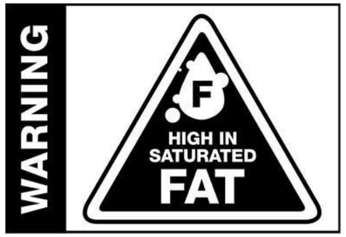

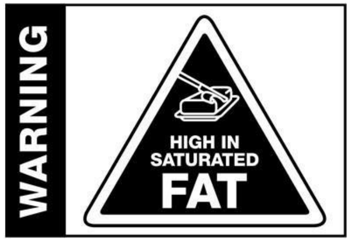

Sugar icons (100% juice)

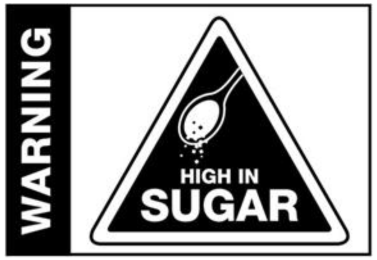

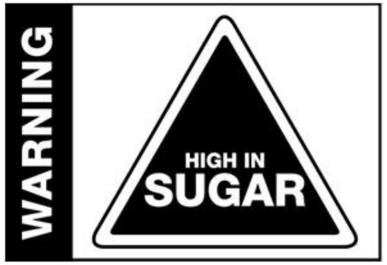

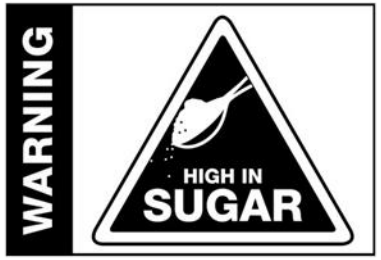

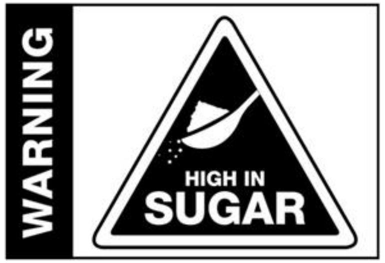

Salt icons (Crisps)

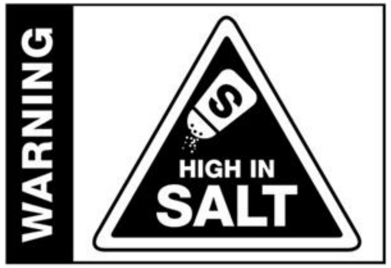

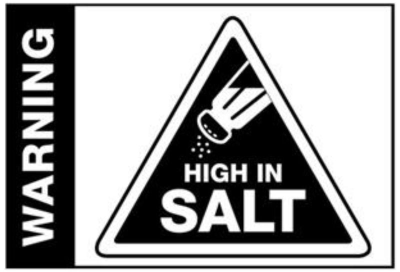

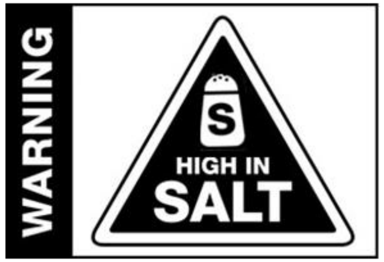

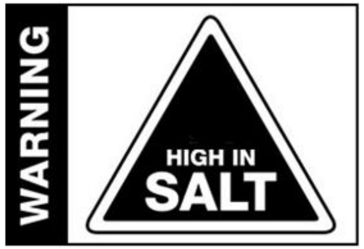

Upper vs Sentence case

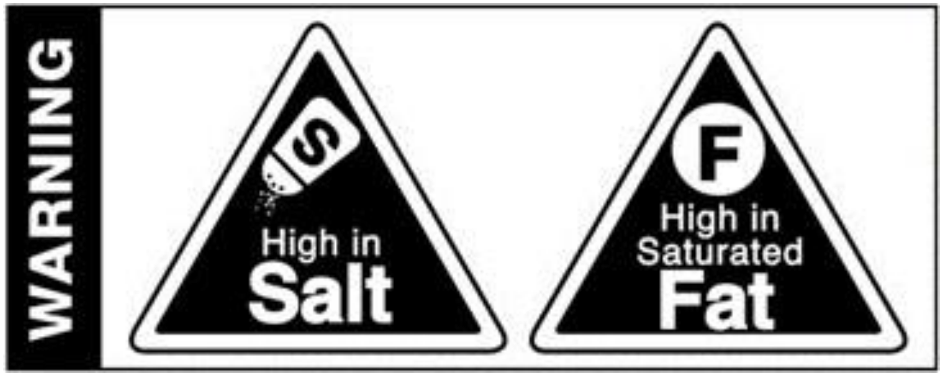

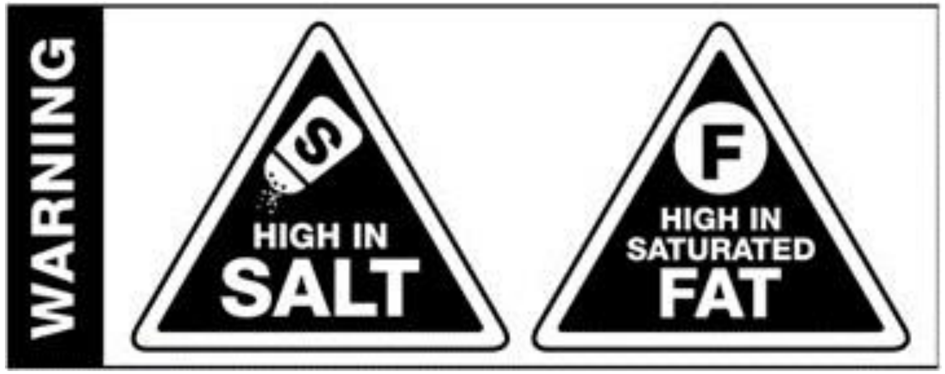

Colour and symbol shapes

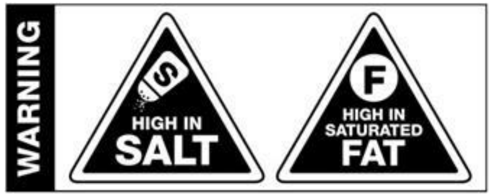

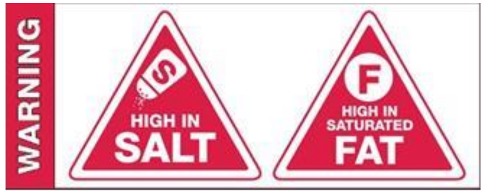

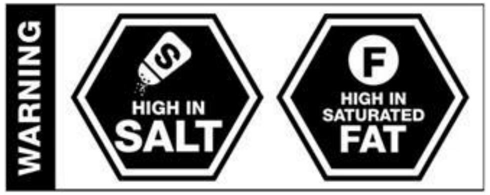

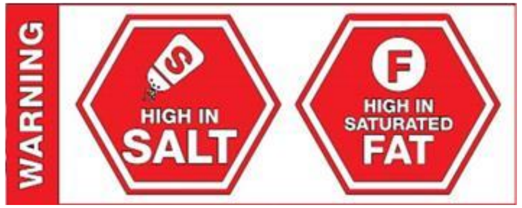

Warning Devices

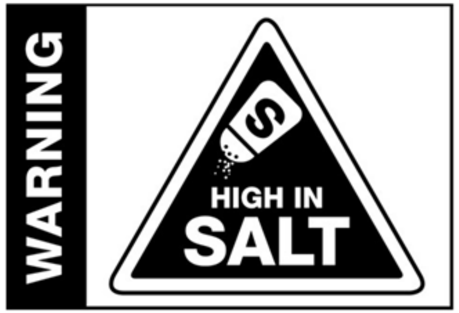

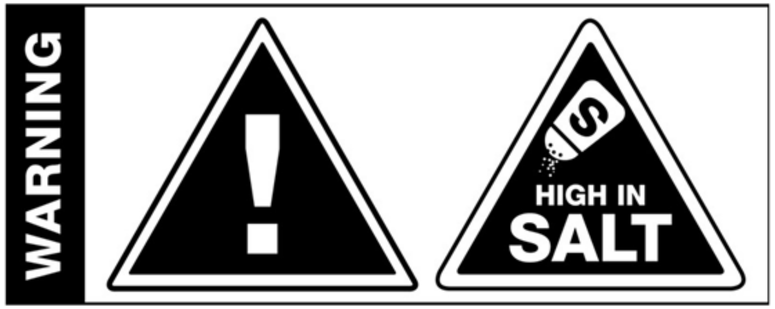

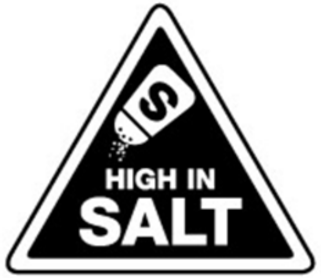

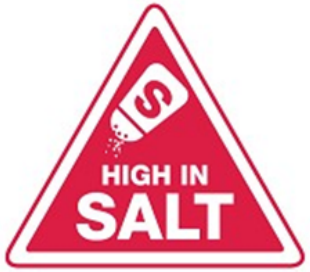

Holding Straps

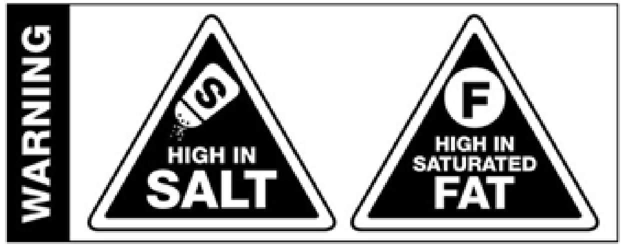

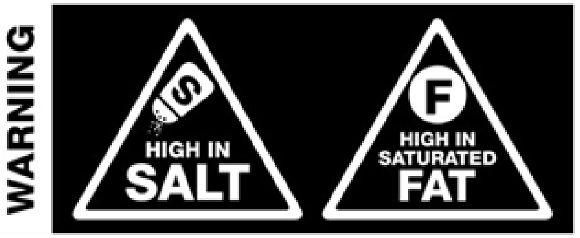

Label Size

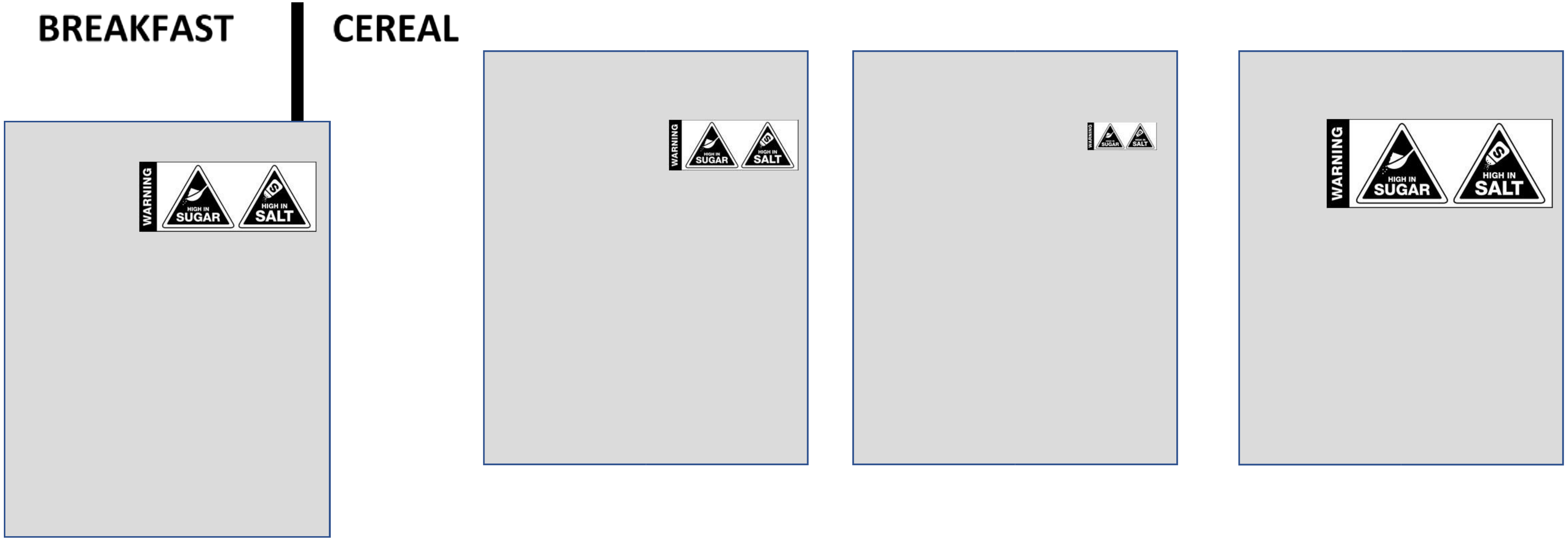

Placement

Top of pack vs

Bottom of pack
