# Supplementary material for: South African consumers’ perceptions of front-of-package warning labels on unhealthy foods and drinks
Source: PLoS One. 2021 Sep 27;16(9):e0257626. doi: 10.1371/journal.pone.0257626 (PMC8475997; doi:10.1371/journal.pone.0257626)
Supplement: S1 File — (PDF) [file pone.0257626.s002.pdf]

Recruitment Questionnaire

Recruitment Questionnaire

Date: Day____________ Month____________ Year: 2019

Time: Hour____________ Minute ____________ AM/PM (please circle)

1	**Location of residence**	☐ Soweto<br>☐ Diepsloot<br>☐ Cosmo city<br>☐ Zanspruit<br>☐ Adams mission<br>☐ Umbumbulu<br>☐ Ndwedwe<br>☐ Durban central<br>☐ Khayelitsha<br>☐ Khayamandi<br>☐ Fisantekraal<br>☐ Gugulethu
2	Do you work in any of the following industries?	☐ Health promotion (thank and terminate)<br>☐ Market Research (thank and terminate)<br>☐ Advertising (thank and terminate)<br>☐ Tobacco industry (thank and terminate)<br>☐ Food and Beverage industry (e.g., supermarkets, restaurants, retail companies) (thank and terminate)<br>☐ None of the above (continue)
3	How often do you purchase branded packaged foods and drinks?<br>*(ensure a mix of participants)*	☐ Never (thank and terminate)<br>☐ Rarely<br>☐ A few times a week<br>☐ About once a day<br>☐ Multiple times a day
4	Are you…	☐ Male<br>☐ Female
5	Are you…	☐ 18 - 35 yrs old<br>☐ 36 - 50 yrs old
6	Please indicate your highest level of education attained:	☐ No Schooling (Not literate)<br>☐ Grade 1 – up to Grade 7 (Low literacy)<br>☐ Passed Grade 7 & above (Literate)
7	Please indicate your household income category<br>*(check quota and ensure a mix of participants)*	☐ No income: R0<br>☐ Low income: R1–R1600<br>☐ Middle income: R1601-R25 600<br>☐ Upper income: R25 601 and above
8	Are you the parent or caregiver for children aged 16 years and below? (*check quotas and ensure a mix of participants*)	☐ Yes<br>☐ No

9	Are you the main decision-maker for food purchases in your home<br><br>*(check quotas and ensure a mix of participants)*	☐ Yes<br>☐ No
10	Are you the main buyer of food and groceries in your home?<br><br>*(check quotas and ensure a mix of participants)*	☐ Yes, I am the main buyer<br>☐ No, but I do share the responsibility<br>☐ No, I am not the main buyer
11	From where do you buy food and groceries most of the time	☐ Loyalty clubs<br>☐ Stokvel clubs<br>☐ Retailers (supermarkets)<br>☐ Retailers (spaza shops, vendors)<br>☐ Other (specify) _______
12	In a typical week, how often do you consume packaged branded foods, such as breakfast cereal, crisps, sweetened beverages, sweets, biscuits, etc<br><br>(*check quotas and ensure a mix of participants*)	☐ Never<br>☐ Rarely<br>☐ A few times a week<br>☐ About once a day<br>☐ Multiple times a day
