# Supplementary material for: South African consumers’ perceptions of front-of-package warning labels on unhealthy foods and drinks
Source: PLoS One. 2021 Sep 27;16(9):e0257626. doi: 10.1371/journal.pone.0257626 (PMC8475997; doi:10.1371/journal.pone.0257626)
Supplement: S2 File — (PDF) [file pone.0257626.s003.pdf]

MODERATOR'S GUIDE

Purpose of the Moderator Guide

This research guide has been developed for the use of the group Moderator for efficient conduct of the research. This guide should be used to steer group discussion to the specified topic areas that need to be covered and the specific questions of interest within each topic area.

Overview of the Moderator's Tasks

The Moderator is required to lead participants through all components of the research. They are to provide instructions, answer questions, and maintain a level of focus and motivation among the group.

Before the study begins, the following need to be done:

1. Labels for testing: Labels for testing will be superimposed onto mockup packs of four different food and drinks packages (*note, four is likely the minimum number of packages required in most countries to test the labels on varied packaging shapes)*. Proposed food packages are chips/crisps (square packet), cereal (rectangular box), yoghurt (yoghurt container) and fruit juice (bottle). An image of the 4 packaged foods/drinks with the label will be projected onto a screen (Appendix D Visual tool). Therefore, just prior to the testing, the moderator should confirm that the audiovisual material, including the order of the presentation, is ready. Prepare a colour printed version of the material to be projected on screen as back-up for each participant (screens are often small in the focus group rooms).
2. Some careful thought should be given to how the respondents are seated for the discussions. They should be seated so that they can all see the material that will be presented to them easily. The seating should also encourage involvement and discussion from all participants in the group. Keep in mind that participants will have to complete rating sheets (Appendix E) independently. Clipboards or small tables will be useful for this aspect.
3. Prepare and check all recording and web casting equipment.
4. Ask participants to read the information sheet (Appendix F) and complete the consent form (Appendix G) as appropriate, including on camera if required by the Biomedical Research Ethics Committee for illiterate participants, and then fill out the short demographics questionnaire (page #2 in the Rating sheet booklet) for use for later analysis, while they wait.
5. The research assistant should be present to help with note taking and to assist participants, without leading or biasing responses, as necessary (for example to assist low literacy participants to fill out the rating exercise).
6. Note, most questions in this moderator guide are qualitative and are to be discussed. A few questions require ratings to be provided; however, for no- literacy groups, rather than provide written ratings, verbal reactions may be sought.
7. Ask all participants to turn off their mobile phones (if they have them).

During the group discussions, the moderator's roles and responsibilities are as follows.

1. The Moderator will need to facilitate open discussion among all participants. They must also manage the duration of the discussion, ensuring that all elements of the label are given the same amount of discussion time, and that the discussion remains focused.
2. It is not necessarily intended that these questions be asked exactly as they are worded here. The discussion should be more like a conversation than a set of structured questions. The discussion should be as informal as possible and participants should be encouraged to speak openly and freely.
3. To the point above, the moderator should set the pace of questions appropriately – even as she probes in all questions in the moderator guide, she may increase or slow down the speed of the discussion based on participants' engagement in the discussion. The moderator will have to exercise own judgment to achieve a balance between thoroughness and avoidance of redundancy in the line of questioning.
4. The Moderator will need to probe with questions such as "Why?" and "What does that mean to you?" in order to fully understand participants' responses.
5. The Moderator will also need to make sure that all participants in the group have an opportunity to express their opinions at different times during the discussion.
6. Because each group of participants may be different, a responsive approach should be used for the research. That is, the moderator should be flexible in their conduct of each group, to allow for individual and group reactions to each element of labels and discussion points. For this reason, the groups may vary in terms of the topics covered and the focus of issues that are discussed.
7. The Moderator and research assistant will need to facilitate the completion of a rating sheet during the session. Please take careful note of moments in the session when ratings ought to be conducted
8. Moderator: The following are the elements of the front of pack warning label that will be tested. Please familiarize yourself with them so that you may guide the participants' effectively.

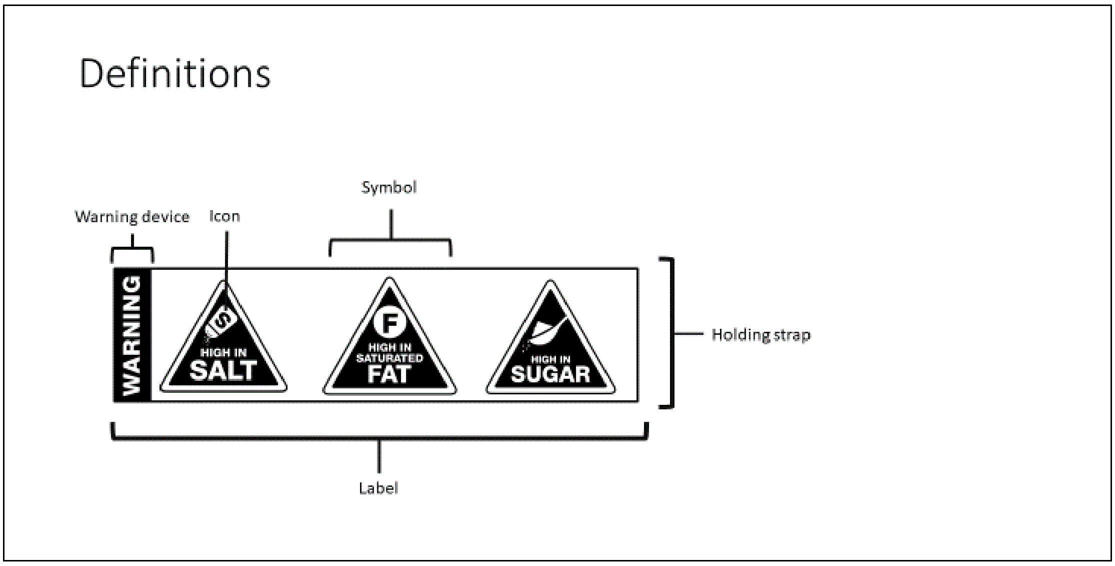

MODERATOR SCRIPT:

1. Introduction and Explanation to Participants:

"Hello, my name is……, I will be conducting the session today, and this is ……, who will assist me by taking notes and helping in the conduct of the research.

Thank you for participating in this study today. We're interested in peoples' reactions to proposed labels for the front of food and drink packages. I will show you a label that is being proposed. I will ask you for your reactions to it.

This study is all about YOUR experiences. There are NO right or wrong answers to anything we discuss today. Having your honest opinion is very important. When you were invited to participate in this discussion today, you already received an information sheet (Appendix F) explaining the purpose of the study as well as the measures that will be taken to ensure that your identify will not be revealed to anyone, and that you may stop participating at any time without negative implications for yourself. Please be assured that your identity will not be revealed to anyone outside this research project. Your answers will be combined with the answers of others so that your identity cannot be known. Therefore, please answer all questions without hesitation. If any of you have questions about the project and your participation, I can answer those questions now. Please also feel free to stop me during the discsussion if you think of another question.

As we are here as part of a group, you will hear information about the others in this room and you will also hear their opinion on the labels that we will show you. To give each one in his room the assurance that their identity and their opinions will not be shared by any of you outside of this room, we also need to agree with each other that we will not share information with others. In front of you, you will find a consent form stating all of this (Appendix G) which you can sign as such an agreement to each other. "Does this sound okay to you? Shall we proceed? " [*Moderator, please note that the consent form must be a loose page – not binded in the booklet with responses – and must be collected before participants proceed to filling in the forms, in order to signal separation of their names/signatures from their responses.]*

"With your permission, we would like to record the group. [*Moderator, if the groups are being viewed from a one-way mirror, or if they are being webcast, please inform the participants of this and assure them of the confidentiality of their participation.*] The recording will only be used to help us with analyzing the results. Your personal details are confidential, and we will not keep or pass on any personal information about you. Is it OK for us to record the group? Can each of you please sign the consent form to indicate your agreement."

[Turn the recording equipment on to record this part of the research – the recording will help with analyzing the results and key points from the discussion.]

[*Moderator: Ensure that all participants have completed the demographic questionnaire on page #2 of the booklet before commencing. If some have not done so yet, then allow them to finish it here before you begin.*]

Warm-up: Participants' introduction

Before we go on to the discussion on labels, it would help us to know a little bit about each of you and for us to get to know one another. Please tell us your name, where do you live, and something about yourself ... I'll start ….

[NOTE to moderator: The goal here is to introduce each person and make everyone comfortable with one another.]

Discussion about the proposed label

Now, I am going to show you an image of some products and then we will discuss what you see. First, I want to make sure that you can all see the image that I project clearly, so I will show you a trial image and once you confirm that you can see it clearly we will proceed to the main image. ***(QUESTIONS HAVE BEEN BEEN REMOVED AS THEY ARE NOT PART OF THIS PAPER AND ALL OTHER QUESTIONS THAT ARE NOT PART OF THIS PAPER HAVE BEEN REMOVED)***

Then, proceed to show the main image for testing. Keep it up for 10 seconds or so until everyone seems to have seen it clearly. Then turn off the image.] [SHOW SLIDE #4]

Visibility/ Memorability

- Did you notice any labels on the packages? Before we discuss it together, in the questionnaire before you, please turn to page #4 [*Moderator, direct participants to the appropriate place in the* booklet]. ***QUESTIONS HAVE BEEN REMOVED AS THEY RE NOT PART OF THIS PAPER***
- "Now, I'm going to project the image back up again and this time I want you to focus closely on a set of labels you will see on the front of the food and drink packages. Study this set of labels closely and I will ask you some questions about it." *[SHOW SLIDE #4]*

[*Moderator: Project the image back again for about 10 seconds. Point to the FoPLs and ensure that all participants have seen it clearly. Once all participants have seen it clearly, turn off the image.]*

[If necessary, reiterate the following: I would like to reiterate that your experience is really important and there is no right and wrong answers. Also, as we are talking about your personal opinions and experiences, it is not necessary for everyone to agree with each other. It is helpful for us to find out the different opinions that people have, as well as where people have the same opinions, so please feel free to tell us whatever you think and feel, even if it might be different to what other people in the room are saying. Also, let's please make sure that only one person speaks at a time. Please allow each person to complete what they are saying.]

Visibility/ Memorability

- Was the labels easily visible? Did it grab your attention? How visible was it? Was it immediately visible or not? Did it catch your eye?
- Was the labels memorable?
- Can you recall it for me now? What exactly did it look like? What do you recall of its shape, color? Was there any text in it? What did it say? [*Without leading their answers, probe respondents' memory of its shape, color, text, icon etc.]*

Comprehensibility

- What do you think is the purpose of these labels?
- What did you understand from the labels?
- Were the labels indicating that the food and drinks were healthy or unhealthy?
- Was it easy to understand? Is there anything you did not understand or that confused you about it?
- Who is it directed to? Do you think it is directed to you? If not you who do you think it is directed to?
- Was it believable?
- Is there anything about this label that is culturally inappropriate? Is there anything about it that is likely to be difficult to understand/interpret in South Africa?

<u>Potential Effectiveness</u>

- Did it change your attitudes towards the food/ drinks that you just saw?
- How did it change attitude, if at all?
- If you were to see this label on packages in a store, would it affect your decision to purchase that product or not? *How* would it affect your purchasing?
- Think of a packaged food or drink that you typically buy. If that product had this label, would it affect your purchase of it? How would it affect your purchase? Would it increase or decrease how often you purchased it?
- In your opinion, what would be the benefits of placing labels like this on unhealthy foods? Whom will they help?
- In your opinion, what are the harms of placing labels like this on unhealthy foods? Whom would they hurt?
- How do you think that labels like this can help/hurt South African society? DO you think they would be effective in addressing the obesity epidemic among adults? … Among children? Why or why not?

<u>Improvements to the label's memorability</u>

- What aspect of the label had the most impact on you and why?
- What was most MEMORABLE in these labels? What image do you remember most? What words do you remember most?
- Was there anything offensive or inappropriate about it?

[NOTE TO RESEARCH PRIMARY INVESTIGATORS: *What follows from this point on in the protocol is a means for testing alternatives to elements in the original label. In the sections that follow, we provide questions to test alternatives to ALL of the label elements. And, we suggest how additional label features may be tested, such as label size and placement. However, note that the execution of all of the following sections may cause the research to be lengthy and redundant. Hence, it is recommended that <u>only alternatives to the most crucial label elements be tested</u>.]*

"Now, we'd like to consider various elements of the label design, and I'd like your views on whether changing it would improve the effectiveness of the label or not. I'll project an image of the original label again and show you the same design with some alterations."

[*Project one of the original images again.*] *[SHOW SLIDE #5]*

"So, here you see one of the original labels again."

[*Move to the next image (slide) which will contain both the original label and the different colored label (by COLOR) in a split screen.*] *[SLOW SLIDE #6]*

"And, here you see both the original label and the altered one. Please turn to page #6 and answer the

questions about these options. **QUESTIONS REMOVED**

The moderator then asks:

- Of the options before you, in your opinion, which label is....”

  - ... more attention-grabbing? Memorable?
  - Which one is more easily visible on a pack?
  - Do the alterations improve your understanding of the label? That is, does it give you a more accurate understanding of what the label intends to say? How does it do so?
  - Does this set of products look more unhealthy or less unhealthy than the original product set? Or are they the same? [*Moderator, show all products together.]*
  - Do the alterations increase or decrease the label’s effectiveness as a warning about the product’s unhealthiness? Why or why not?
  - Do the alterations increase or decrease your understanding of the food’s healthfulness? Why or why not?
  - Which colour do you remember most?
  - Do the alterations increase or decrease your intent to purchase the product? Why or why not?
  - Is there anything inappropriate or offensive about the alternative proposed?
  - Of the options before you, which one do you think is more likely to be effective in grabbing people’s attention and deterring the purchase of an unhealthy food?

“Now, I’d like to look at the label even more closely. This time, I’d like you to consider the ICONS or the pictures we’ve used in the label. I’ll show you one of the original labels again, and an alternative to the original.”

[*Moderator to move to the appropriate slides, in step with the discussion and questions below.]*

“Let’s start with the icon of FAT. Here’s one of the the original labels with the icon originally used to depict fat. [SHOW SLIDE #7] And now here are some other ways in which we could depict fat. [*Moderator to move to the appropriate slides, in step with the discussion and questions.] [SHOW SLIDE #8]* Please turn to page #7 and answer the questions about these options. ***QUESTIONS REMOVED***

The moderator then asks:

- Of the options before you, which one is:
  - ... more attention-grabbing? Memorable?
  - Which one is more easily visible on a pack?
  - Do the alterations improve your understanding of the label? That is, does it give you a more accurate understanding of what the label intends to say? How does it do so?
  - Does any of the alternative icons look more unhealthy or less unhealthy than the original product icon? Or are they the same? [*Moderator, show all products together SLIDE #8]*
  - Do the alterations increase or decrease the label’s effectiveness as a warning about the product’s unhealthiness? Why or why not?
  - Do the alterations increase or decrease your understanding of the food’s healthfulness? Why or why not?
  - Do the alterations increase or decrease your intent to purchase the product? Why or

why not?
- ➢ Is there anything inappropriate or offensive about the alternative proposed?
- ➢ Of the options before you, which one do you think is more likely to be effective in grabbing people's attention and deterring the purchase of an unhealthy food?

Now let's move to the icon of SUGAR. Here's the original label with the icon originally used to depict sugar *[SHOW SLIDE #9]*. And now here are some other ways in which we could depict sugar." [*Moderator to move to the appropriate slides, in step with the discussion and questions.] [SHOW SLIDE #10]* Please turn to page #8 and answer the questions about these options.

[Moderator, please direct participants to complete the short rating exercise of the comparison. Once completed, participants should move to discussing the alternatives. Once all participants have concluded the rating, proceed ...]

- Of the options before you, which one is:
  - ➢ ... more attention-grabbing? Memorable?
  - ➢ Which one is more easily visible on a pack?
  - ➢ Do the alterations improve your understanding of the label? That is, does it give you a more accurate understanding of what the label intends to say? How does it do so?
  - ➢ Does any of the alternative icons look more unhealthy or less unhealthy than the original product icon? Or are they the same? [*Moderator, show all products together SLIDE #10]*
  - ➢ Do the alterations increase or decrease the label's effectiveness as a warning about the product's unhealthiness? Why or why not?
  - ➢ Do the alterations increase or decrease your understanding of the food's healthfulness? Why or why not?
  - ➢ Do the alterations increase or decrease your intent to purchase the product? Why or why not?
  - ➢ Is there anything inappropriate or offensive about the alternative proposed?
  - ➢ Of the options before you, which one do you think is more likely to be effective in grabbing people's attention and deterring the purchase of an unhealthy food?

Now let's move to the icon of SALT. Here's the original label with the icon originally used to depict salt [SHOW SLIDE #11]. And now here are some other ways in which we could depict salt." [*Moderator to move to the appropriate slides, in step with the discussion and questions.]* [SHOW SLIDE #12] Please turn to page #9 and answer the questions about these options. ***QUESTIONS REMOVED***

The moderator then asks:

- Of the options before you, which one is:
  - ➢ ... more attention-grabbing? Memorable?
  - ➢ Which one is more easily visible on a pack?
  - ➢ Do the alterations improve your understanding of the label? That is, does it give you a more accurate understanding of what the label intends to say? How does it do so?
  - ➢ Does any of the alternative icons look more unhealthy or less unhealthy than the original product icon? Or are they the same? [*Moderator, show all products together SLIDE #12]*
  - ➢ Do the alterations increase or decrease the label's effectiveness as a warning about the product's unhealthiness? Why or why not?
  - ➢ Do the alterations increase or decrease your understanding of the food's healthfulness?

Why or why not?
- ➢ Do the alterations increase or decrease your intent to purchase the product? Why or why not?
- ➢ Is there anything inappropriate or offensive about the alternative proposed?
- ➢ Of the options before you, which one do you think is more likely to be effective in grabbing people's attention and deterring the purchase of an unhealthy food?

"Now, I'd like us to look again at the ICON we've used in the labels. I'll show you the original with and without the ICON." [SHOW SLIDE #13]

[*Moderator to move to the appropriate slides, in step with the discussion and questions below.]*

Here's the original label with the icon originally used to depict salt, sugar and fat. And now here it is without those accompanying icons." [*Moderator to move to the appropriate slides, in step with the discussion and questions. Moderator will reitereate to group that we are just talking about the icons and the two products displayed are just as an example.]* Please turn to page #10 and answer the questions about these options. ***QUESTIONS REMOVED***

The moderator then asks:

- Of the options before you, which one is ...:
  - ➢ ... more attention-grabbing? Memorable?
  - ➢ Which one is more easily visible on a pack?
  - ➢ Do the alterations improve your understanding of the label? That is, does it give you a more accurate understanding of what the label intends to say? How does it do so?
  - ➢ Does this set of products look more unhealthy or less unhealthy than the original product set? Or are they the same? [*Moderator, show all products together SLIDE #13.]*
  - ➢ Do the alterations increase or decrease the label's effectiveness as a warning about the product's unhealthiness? Why or why not?
  - ➢ Do the alterations increase or decrease your understanding of the food's healthfulness? Why or why not?
  - ➢ Do the alterations increase or decrease your intent to purchase the product? Why or why not?
  - ➢ Is there anything inappropriate or offensive about the alternative proposed?
  - ➢ Of the options before you, which one do you think is more likely to be effective in grabbing people's attention and deterring the purchase of an unhealthy food?

"Now, I'd like us to look at the TEXT we've used in the label. I'll show you the original again, and alternatives to the original."

[*Moderator to move to the appropriate slides, in step with the discussion and questions below.]*

- "Here's the original TEXT and here are alternatives *[SHOW SLIDE #14]*. [*Moderator to move to the appropriate slides, in step with the discussion and questions.]* Please turn to page #11 and answer the questions about these options. ***QUESTIONS REMOVED***

The moderator then asks:

- Of the options before you, which one is …:
  - … more attention-grabbing? Memorable?
  - Which one is more easily visible on a pack?
  - Do the alterations improve your understanding of the label? That is, does it give you a more accurate understanding of what the label intends to say? How does it do so?
  - Does this set of products look more unhealthy or less unhealthy than the original product set? Or are they the same? [*Moderator, show all products together SLIDE #14.]*
  - Do the alterations increase or decrease the label's effectiveness as a warning about the product's unhealthiness? Why or why not?
  - Do the alterations increase or decrease your understanding of the food's healthfulness? Why or why not?
  - Do the alterations increase or decrease your intent to purchase the product? Why or why not?
  - Is there anything inappropriate or offensive about the alternative proposed?
  - Of the options before you, which one do you think is more likely to be effective in grabbing people's attention and deterring the purchase of an unhealthy food?

"Now, I'd like us to look at the SYMBOL/HOLDING SHAPE we've used for the label. [*Moderator, point to the outside shape to ensure that participants have understood exactly what was meant.]* Here's the original label. And now here is an alternative shape." [SHOW SLIDE #15] [*Moderator to move to the appropriate slides, in step with the discussion and questions below.] "*Please turn to page #12 and answer the questions about these options." ***QUESTIONS REMOVED.***

The moderator then asks:

- Of the options before you, which one is …:
  - … more attention-grabbing? Memorable?
  - Which one is more easily visible on a pack?
  - Do the alterations improve your understanding of the label? That is, does it give you a more accurate understanding of what the label intends to say? How does it do so?
  - Does this set of products look more unhealthy or less unhealthy than the original product set? Or are they the same? [*Moderator, show all products together.]*
  - Do the alterations increase or decrease the label's effectiveness as a warning about the product's unhealthiness? Why or why not?
  - Do the alterations increase or decrease your understanding of the food's healthfulness? Why or why not?
  - Do the alterations increase or decrease your intent to purchase the product? Why or why not?
  - Is there anything inappropriate or offensive about the alternative proposed?
  - Of the options before you, which one do you think is more likely to be effective in grabbing people's attention and deterring the purchase of an unhealthy food?

"Now, I'd like us to look at the WARNING DEVICES in the label. [*Moderator, ensure that the participants understand what is being referred to.*] I'll show you the original again, and an alternative to the original." [*Moderator to move to the appropriate slides, in step with the discussion and questions below.] [SHOW SLIDE #16]*

"Here's the original label with the original warning device. And now here are two alternatives; one

with an additional exclamation mark and one without the warning text. [*Moderator to move to the appropriate slides, in step with the discussion and questions.] "*Please turn to page #13 and answer the questions about these options." ***QUESTIONS REMOVED***

The moderator then asks:

- Of the options before you, which one is …:
  - … more attention-grabbing? Memorable?
  - Which one is more easily visible on a pack?
  - Do the alterations improve your understanding of the label? That is, does it give you a more accurate understanding of what the label intends to say? How does it do so?
  - Does this set of products look more unhealthy or less unhealthy than the original product set? Or are they the same? [*Moderator, show all products together.]*
  - Do the alterations increase or decrease the label's effectiveness as a warning about the product's unhealthiness? Why or why not?
  - Do the alterations increase or decrease your understanding of the food's healthfulness? Why or why not?
  - Do the alterations increase or decrease your intent to purchase the product? Why or why not?
  - Is there anything inappropriate or offensive about the alternative proposed?
  - Of the options before you, which one do you think is more likely to be effective in grabbing people's attention and deterring the purchase of an unhealthy food?

"Now, I'd like us to look at the HOLDING STRAP we've used around the label. [*Moderator, ensure that the participants have understood what is referred to.*] I'll show you the original again, and an alternative to the original." [SHOW SLIDE #17]

[*Moderator to move to the appropriate slides, in step with the discussion and questions below.]*

"Here's the original (white). And now here is an alternative (black). *"*Please turn to page #14 and answer the questions about these options." ***QUESTIONS REMOVED***

The moderator then asks:

- Of the options before you, which one is … :
  - … more attention-grabbing? Memorable?
  - Which one is more easily visible on a pack?
  - Do the alterations improve your understanding of the label? That is, does it give you a more accurate understanding of what the label intends to say? How does it do so?
  - Does this set of products look more unhealthy or less unhealthy than the original product set? Or are they the same? [*Moderator, show all products together.]*
  - Do the alterations increase or decrease the label's effectiveness as a warning about the product's unhealthiness? Why or why not?
  - Do the alterations increase or decrease your understanding of the food's healthfulness? Why or why not?
  - Which colour do you remember most?
  - Do the alterations increase or decrease your intent to purchase the product? Why or why not?
  - Is there anything inappropriate or offensive about the alternative proposed?
  - Of the options before you, which one do you think is more likely to be effective in grabbing people's attention and deterring the purchase of an unhealthy food?

"Now, moving on, I'd like us to consider how different SIZES of the label may improve or reduce its effectiveness."

[*Moderator to move to the appropriate slides, in step with the discussion and questions below.]* *[SHOW SLIDE #18]* "Please turn to page #15 and answer the questions about these options." ***QUESTIONS REMOVED***

The moderator then asks:

"Here's the original label. And now here are alternatives in different sizes

- Of the options before you, which one is …:
  - … more attention-grabbing? Memorable?
  - Which one is more easily visible on a pack?
  - Do the alterations improve your understanding of the label? That is, does it give you a more accurate understanding of what the label intends to say? How does it do so?
  - Does this set of products look more unhealthy or less unhealthy than the original product set? Or are they the same? [*Moderator, show all products together.]*
  - Do the alterations increase or decrease the label's effectiveness as a warning about the product's unhealthiness? Why or why not?
  - Do the alterations increase or decrease your understanding of the food's healthfulness? Why or why not?
  - Do the alterations increase or decrease your intent to purchase the product? Why or why not?
  - Is there anything inappropriate or offensive about the alternative proposed?
  - Of the options before you, which one do you think is more likely to be effective in grabbing people's attention and deterring the purchase of an unhealthy food?

"Now, moving on, I'd like us to consider how PLACEMENT of the label on the front of the package may improve or reduce its effectiveness." [SHOW SLIDE #19] [*Moderator to move to the appropriate slides, in step with the discussion and questions below.]* "Please turn to page #16 and answer the questions about these options." ***QUESTIONS REMOVED***

The moderator then asks:

"Here's the original label. And now here are an alternative placement position.

- Of the options before you, which one is…:
  - … more attention-grabbing? Memorable?
  - Which one is more easily visible on a pack?
  - Do the alterations improve your understanding of the label? That is, does it give you a more accurate understanding of what the label intends to say? How does it do so?
  - Does this set of products look more unhealthy or less unhealthy than the original product set? Or are they the same? [*Moderator, show all products together.]*
  - Do the alterations increase or decrease the label's effectiveness as a warning about the product's unhealthiness? Why or why not?
  - Do the alterations increase or decrease your understanding of the food's healthfulness? Why or why not?
  - Do the alterations increase or decrease your intent to purchase the product? Why or

why not?

- Is there anything inappropriate or offensive about the alternative proposed?
- Of the options before you, which one do you think is more likely to be effective in grabbing people's attention and deterring the purchase of an unhealthy food?

Energy ***QUESTIONS REMOVED***

Comparative Rating

Now, thinking of all the labels you've seen today, does any **one** label stand out for you? Which one would that be?

General Discussion **QUESTIONS REMOVED**
