# Supplementary material for: South African consumers’ perceptions of front-of-package warning labels on unhealthy foods and drinks
Source: PLoS One. 2021 Sep 27;16(9):e0257626. doi: 10.1371/journal.pone.0257626 (PMC8475997; doi:10.1371/journal.pone.0257626)
Supplement: S1 Table — (PDF) [file pone.0257626.s004.pdf]

S1 Table. Themes, subthemes and quotes

Themes	Subthemes	Quotes
Positive attitude toward warning labels		"It is helpful;, look now we have all these ailments because we just eat anything and everything" (female, low income, low literacy, urban).
Perceived benefits of warning labels	Warn of health implications	"Yes, it shifts your focus from just seeing nice chips to the health hazards on them" (female, low income, low literacy, urban).
	Provide useful nutrition information	"Because it is very simple, it is saying that there is too much salt and therefore I must not buy it" (female, middle-high income, literate, urban).
	Educational	"It does (help) because normally we would buy juice because it is considered healthy and now we know how to check for the levels of sugar in the juice; we now know how to check for levels of salt as well. We are well knowledgeable now (female, low income, no literacy, rural).

	Easily understandable	"The labels can be easily understood by the less literate, young people, and the elderly. The sign of a spoon full of sugar, even if you do not know how to read, it makes it easy to understand this is sugar. And the salt is easy to identify since we use it when laying the table" (female, middle-high income, literate, rural).
	Benefit child health	"When I send a child to buy something, they will see the sign and know whether that this is good or not" (male, low income, no literacy, urban).
	Provide succinct information	"I also believe the reason why they came up with the triangle concept for the fat warning and salt is because in most cases you find that at the back they tell you about the kilojoules, so that small triangle you read fast unlike going through the whole nutritional table like the one we have now. I believe it is going to make our lives easier" (female, low income, low literacy, urban).
Perceived behavior modification	Cautiousness	"The label will tell me but that will not necessarily stop me from buying the product. I might be influenced to buy it less often but

		not to entirely disuse the product, particularly if it is something I love" (female, low income, no literacy, rural).
	Indifference toward warning labels	"It is like the cigarette problem, cigarettes are written 'Dangerous: smoking can kill you,' but smokers still smoke" (female, middle-high income, literate, urban).
Positive elements of warning labels	Visibility	When you buy the yogurt, you can easily see the label" (male, low income, no literacy, urban).
	Color	"Even the yogurt container has bright blue and pink colors, and the black signs make you want to know what is written there" (female, middle-high income, literate, urban).
	Position	"This label is right because it is placed in front, people will be able to notice before taking the product" (female, middle-high income, literate, rural).
	Text	"It took me time until I saw the word 'warning,' then that shook me a bit (female, low income, low literacy, urban).

	Emphasis	"[An exclamation mark] is a sign of warning and emphasis" (male, low income, no literacy, urban).
	Symbols	"It has so many signs and it will direct your eyes to the product for a longer time because you would be wondering why it has so many signs of warning" (male, low income, no literacy, urban).
